# Recent Advances and Potential Multi-Omics Approaches in the Early Phases of Inflammatory Bowel Disease

**DOI:** 10.3390/jcm12103418

**Published:** 2023-05-11

**Authors:** Iago Rodríguez-Lago, Jonathan Blackwell, Beatriz Mateos, Urko M. Marigorta, Manuel Barreiro-de Acosta, Richard Pollok

**Affiliations:** 1 Gastroenterology Department, Hospital Universitario de Galdakao, 48960 Galdakao, Spain; 2 Biocruces Bizkaia Health Research Institute, 48960 Galdakao, Spain; 3 Deusto University, 48007 Bilbao, Spain; 4 Gastroenterology, Western General Hospital, Edinburgh EH4 2XU, UK; 5 Integrative Genomics Lab, Center for Cooperative Research in Biosciences (CIC bioGUNE), Basque Research and Technology Alliance (BRTA), Bizkaia Technology Park, 48160 Derio, Spain; 6 IKERBASQUE, Basque Foundation for Sciences, 48009 Bilbao, Spain; 7 Gastroenterology Department, Hospital Clínico Universitario de Santiago, 15706 Santiago de Compostela, Spain; 8 Gastroenterology Department, St George’s University of London, London SW17 0RE, UK

**Keywords:** Crohn’s disease, ulcerative colitis, diagnosis, early, preclinical

## Abstract

Inflammatory bowel disease leads to debilitating gastrointestinal symptoms and reduced quality of life, resulting in a significant burden on healthcare utilization and costs. Despite substantial advancements in diagnosis and treatment, there may still be considerable delays in diagnosing some patients. To reduce disease progression before the full disease spectrum appears and improve prognostic outcomes, several strategies have concentrated on early intervention and prevention. Recent evidence shows that initial immune response changes and endoscopic lesions may exist for years before diagnosis, implying the existence of a preclinical phase of inflammatory bowel disease comparable to findings in other immune-mediated disorders. In this review, we highlight the most relevant findings regarding preclinical inflammatory bowel disease and the prospective role of novel omics techniques in this field.

## 1. Introduction

Inflammatory bowel disease (IBD) is a chronic immune-mediated disorder with a relapsing and remitting course. This term includes two main subtypes: Crohn’s disease (CD) and ulcerative colitis (UC). The prevalence of IBD is rapidly increasing worldwide, and it leads to disabling gastrointestinal symptoms, low quality of life, and a significant burden for healthcare utilization and associated costs [1].

Its pathophysiology has still not been fully determined, but most recent evidence suggests that the diagnosis is the end result of a prolonged process where a variety of environmental risk factors interact with at-risk individuals. Despite recent advances, their exact role and the duration of this period still remains unknown. Most recent data suggest that an altered immune response and even endoscopic lesions can be present even years before the diagnosis, similar to what has been described in other immune-mediated diseases like rheumatoid arthritis, systemic lupus erythematosus, or diabetes mellitus type 1 [2]. These new findings warrant a new perspective that incorporates novel strategies for earlier intervention in order to improve prognosis by reducing the risk of bowel damage and complicated disease phenotype [3,4].

Although evidence of the initial (pre-symptomatic) phases of IBD is still limited, promising data on a wide range of biomarkers and altered cellular pathways have recently shown that opportunities for achieving a timely diagnosis are available [5]. In the future, goals for improving the management of IBD will include achieving better patient stratification and application of individualised management [6]. In view of the increasing burden of IBD and its impact on quality of life, the most significant benefits will likely result from the identification of subjects at higher risk of developing the disease and its complications (prediction) and the development of disease-modification strategies that could potentially halt the development of IBD (prevention). This review provides a comprehensive review of the most recent data on the preclinical phase of IBD and identifies unmet needs for future research in this area.

## 2. The Concept of Preclinical Disease

### 2.1. Diagnosis of IBD and Impact of Diagnostic Delay

The diagnostic journey of a person living with IBD typically starts with gastrointestinal symptoms that eventually lead to investigations including colonoscopy and imaging of the abdomen. However, diagnosing IBD can be challenging, as typical presenting symptoms of abdominal pain, diarrhoea, or rectal bleeding are non-specific, and roughly a quarter of the non-IBD population present to primary care centres with gastrointestinal symptoms at some point over a 10-year period [7]. These symptoms collectively have less than a 4% positive predictive value for IBD, presenting clinicians with difficulty in determining which individuals should be sent for costly and, in some cases invasive, investigations such as colonoscopy, magnetic resonance enterography, and/or video capsule endoscopy [8].

Faecal calprotectin, a highly sensitive and non-invasive biomarker of inflammation within the gut, plays an important role in risk stratifying patients for further investigation, as 98% of those with a calprotectin of <100 µg/g will not eventually be diagnosed with IBD [9]. Unfortunately, calprotectin testing remains underutilised in primary care, as was demonstrated in a population-based study in the United Kingdom, which found only 3.1% of patients with appropriate symptoms had their calprotectin levels measured [10]. Therefore, effective triage of referrals for suspected IBD is difficult, resulting in more than half of patients with undiagnosed IBD waiting at least 18 months before receiving specialist review, with longer delays for individuals with previous diagnostic labels of irritable bowel syndrome and depression [7]. As a consequence, some individuals are not investigated promptly and are subject to a delayed diagnosis of IBD.

It is well established that over time, active CD can result in cumulative bowel damage, resulting in strictures and fistulae, while in ulcerative colitis there is an increased risk of colorectal cancer [11]. Early initiation of thiopurines and tumour necrosis factor (TNF) inhibitors can potentially act as disease-modifying agents in CD by reducing the risk of complications, while observational studies support a potential chemo-preventative effect of mesalamine in reducing the risk of colorectal cancer in UC [12,13,14,15]. Diagnostic delay by definition deprives patients of early treatment and its potential benefits. A recent meta-analysis by Jayasooriya et al. demonstrated that individuals with CD whose time to diagnosis from symptom onset was above the 75th percentile of the longest duration (median 24 months) were more likely to have stricturing or penetrating disease and were twice as likely to have intestinal surgery relative to those with a shorter time to diagnosis, while in UC, diagnostic delay was associated with increased likelihood of colectomy [4]. Therefore, reducing diagnostic delay should be one of the first steps in early intervention strategies.

### 2.2. Evidence of Symptoms before Diagnosis of IBD

The clinical presentation of IBD varies significantly between patients, depending on disease phenotype and location [16]. Individuals with UC usually experience bloody diarrhoea, urgency, and sometimes abdominal pain; CD can present in a similar fashion, particularly if affecting the colon, but more often presents with non-bloody diarrhoea, perianal pain or discharge, fever, weight loss or intestinal obstruction causing abdominal distention, and vomiting. To further complicate their assessment, before being diagnosed with IBD, the first manifestation of the disease can be extraintestinal in 7.5% or more of patients, most commonly peripheral arthritis and ankylosing spondylitis [17], and a small proportion of patients may even be asymptomatic when incidentally diagnosed with IBD at colonoscopy screening programmes for bowel cancer [18,19].

The median time from symptom onset to diagnosis of CD and UC is 8 and 3.7 months, respectively [4]. However, there is evidence of excess gastrointestinal symptoms in the IBD population for up to 10 years before diagnosis [7] and this is mirrored by increased healthcare utilisation and costs during this period [20,21,22]. A large case-control study of IBD patients found them to be three times as likely to have received a diagnosis of irritable bowel syndrome in the period before IBD diagnosis compared with matched controls during the equivalent period [23]. In some cases, the label of irritable bowel syndrome may represent a misdiagnosis and delay diagnosis of established IBD, yet the possibility remains that there might exist a symptomatic prodrome to IBD before mucosal inflammation develops and the disease is detectable at endoscopy. This hypothesis has been explored within the Swedish nationwide histology cohort, where Sun et al. demonstrated that patients who have normal histology on their first ileocolonic biopsies have a subsequent increased risk of later being diagnosed with IBD compared with matched controls, suggesting the possibility that gastrointestinal symptoms may have prompted investigation with colonoscopy and biopsy but IBD was not yet detectable even histologically [24]. Therefore, there is an urgent need to reduce the diagnostic delay by increasing awareness of the disease in the general population and by improving referral pathways.

### 2.3. Defining Preclinical IBD

The current consensus on the definition of early CD has led to the development of recommendations on new therapeutic targets (STRIDE-II) and endpoints (SPIRIT) for future clinical trials [25,26]. However, the most recent evidence has demonstrated that patients with IBD undergo a period that precedes the development of symptoms and can be detected in asymptomatic individuals, defined as the preclinical phase [2,5]. The occurrence of this subclinical period where the initial immunological disturbances are already detectable is of great interest for identifying the earliest stages of the disease and fully characterising the natural history of both CD and UC. Furthermore, this will open the discussion on what the aim of medical therapy at this stage where the full (symptomatic) spectrum of the disease has not developed yet should be.

These new findings have emerged alongside the approval of new drugs targeting different pathways in both diseases. While such treatments have shown efficacy in controlling the inflammatory cascade in most cases, concerns have arisen about their long-term efficacy [27] and the potential for reversing complicated lesions (i.e., strictures or fistulas) and halting bowel damage at later stages [28,29,30,31]. It is now widely recognized that heterogeneous pathogenic routes can be involved in each patient, explaining the variability of the phenotypic and clinical manifestations of IBD alongside the potential evolution of the disease over time [32,33].

**Figure 1:**
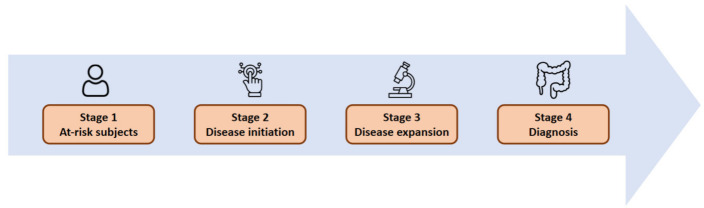
Proposed stages of the early events preceding the diagnosis of IBD, as suggest by Torres et al. [5].

This rationale has led to increased interest in identifying individuals at higher risk of developing IBD and evaluating different ways of non-invasive assessment. Thus, a recent expert consensus has established the stages into which the period of time before the final diagnosis, known as the preclinical phase, can be divided (Figure 1) [5].

Multiple events at different levels have been described during this four-stage process. Interestingly, different cohorts focused on the early phases of the disease have found a range of circulating antibodies (anti-microbial [34,35,36], anti-GM-CSF [37], anti-integrin αvβ6 [38]), serum protein signatures [35,39,40,41], metabolomic features [42], microbiome signatures [43], gut barrier dysfunction [44,45], faecal markers [46], cellular pathways [39], and mucosal lesions [19] that clearly delineate this period where the disease is still silent, highlighting that timely identification strategies are available, at least in high-risk individuals (Table 1).

### 2.4. Subclinical Bowel Lesions

Apart from circulating biomarkers, the detection of mucosal abnormalities in otherwise healthy subjects, particularly in the context of colorectal cancer screening programs, have also unveiled a new subgroup of patients in whom preclinical disease can be characterized [18,19,49,50,51,52,53,54,55,56,57]. The prevalence of these findings within population-based screening programs is approximately 0.35%, and mostly includes cases of UC [19]. Observational studies have also provided information on the prognosis of the disease in this context, determining that around one-third of these patients will develop symptomatic disease after a median follow-up of 25 months [19], and this number can progressively increase up to 58% after a median of 7 years [22]. One of the main advantages of these cohorts is the availability of endoscopic data and tissue samples of the initial bowel lesions that can reveal novel tissue biomarkers of early disease [49]. In addition to identifying them, the characterisation of patients with subclinical endoscopic activity can also help to shape a more individualised management of the disease in the future.

While patients with an incidental diagnosis are typically asymptomatic, they may still require medical intervention during the pre-diagnostic period, leading to increased costs and decreased work productivity [20,21,22]. Subclinical lesions have recently been associated with an increase in the number of visits to primary care facilities and the use of steroids up to 5 years before diagnosis compared with matched non-IBD healthy controls [22]. Interestingly, as the disease advances to its symptomatic debut, access to healthcare resources changes towards an increase in the number of visits to primary care, specialist care, emergency hospital attendance, and hospitalisation, and is associated with more radiological examinations, sick leave, and use of antibiotics and even systemic steroids [22,58]. However, the exact mechanisms and causes leading to these findings are unclear. Whether its gut-associated symptoms or systemic consequences (e.g., fatigue) are the drivers for seeking medical assistance will need to be explored in the future. Through a better understanding of the impact of these lesions, healthcare professionals will be able to develop strategies targeting disease progression at an early stage. In addition, future research in this field will likely involve multi-omics studies with longer follow-ups in order to provide a more detailed characterisation of patients with subclinical endoscopic activity. Ongoing trials (ClinicalTrials.gov: NCT05698745) will be addressing this question from a multidisciplinary perspective. Integrating these datasets will not only help to better understand the pathophysiology of the disease but also identify potential targets for future treatments.

### 2.5. Medical Intervention in Early IBD

The development of prognostic tools to predict disease outcomes in IBD offers the opportunity to tailor treatment to the individual, the so-called “precision medicine”. The paradigm that there is a “window of opportunity” where early “top-down” initiation of potent therapy can alter disease progression has evolved. Evidence supporting this approach does exist. Initiation of thiopurines and TNF inhibitors may delay disease progression to a stricturing or penetrating disease phenotype [59]. Early treatment with both thiopurine and TNF inhibitors may achieve disease modification. Early thiopurine use may slow disease progression and reduce the requirement for intestinal and perianal surgery in certain cohorts [60,61,62,63]. Likewise, post hoc analysis of the Crohn’s Trial of the Fully Human Antibody Adalimumab for Remission Maintenance (CHARM) found patients with a disease duration of less than 2 years at the time of initiating adalimumab had higher rates of remission compared with those starting later [64]. Observational data also indicate that very early anti-TNF treatment is associated with reduced requirement for CD-related surgery [65,66,67].

Unlike CD, evidence of the benefit of early treatment of UC in altering clinical outcomes is sparse and inconclusive. A recent meta-analysis found that delayed diagnosis is associated with an increased risk of colectomy; however, only two studies were eligible for inclusion [4]. An individual patient data meta-analysis found no difference in treatment outcomes based on the duration of UC [68]. Recent findings from the epi-IIRN report that early initiation of biologics is associated with better clinical outcomes in CD but not UC [69]. Therefore, further evidence on the impact of early intervention in UC is needed. While long-term complications are more clearly defined in CD, these should also be considered with respect to UC [70]. Disease modification strategies may thus improve outcomes in a proportion of IBD patients.

Both UC and CD may follow a variable disease course ranging from an indolent to an aggressive, rapidly progressing pattern of behaviour. Thus, early treatment with potent biologic agents, small molecules, or immunomodulators is not appropriate for all patients with a risk of overtreatment and consequent potential adverse events associated with these agents in those with mild disease. Prognostic tools may allow stratification of patients who are most likely to benefit from an early “Top Down” therapeutic approach [6].

Some clinical features are predictive of prognosis in both CD and UC [71,72]. In CD, clinical predictors of “complicated” disease outcomes include extensive small bowel disease, perianal disease, early stricturing/penetrating disease, smoking, and young age at diagnosis. In UC, they include young age, male sex, extensive colitis, severe disease activity at diagnosis, and early steroid use. However, validation studies have not confirmed their use as a decision-making tool in clinical practice [73].

## 3. Tools for IBD Prediction

### 3.1. Circulating Biomarkers

**Figure 2:**
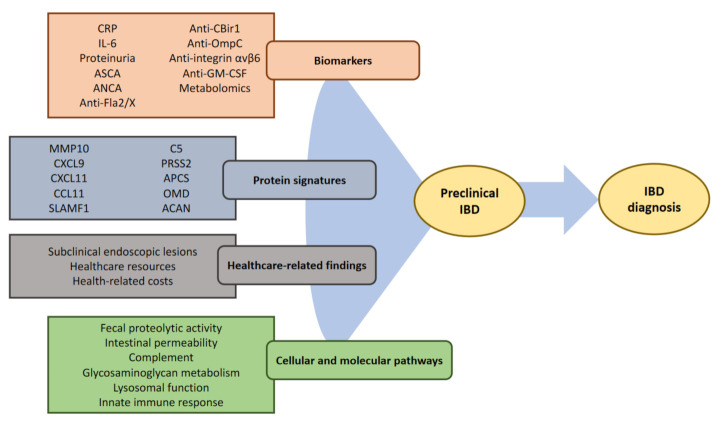
Overview of clinical and omics findings in preclinical IBD.

Several serological markers have been identified that are present prior to the diagnosis of IBD, mainly through the presence of multiple anti-microbial antibodies (Figure 2, Table 1). These antibodies have also been detected in healthy first-degree relatives, with ASCA, pANCA, and anti-OmpC being more frequently reported, while ACCA, ALCA, and anti-flagellin I2 and CBir1 have also been found in some reports [74,75,76]. The presence of these antibodies highlights the humoral immune dysregulation in at-risk populations, which may be linked to dysbiosis in some of these individuals. The United States Department of Defense Serum Repository has revealed that these circulating antibodies are also present years before developing CD [34]. In this study, up to 65% of samples tested positive for at least one antibody on the first available sample, and anti-microbial antibody positivity was associated with a complicated disease course, probably reflecting a longer period of time with active subclinical inflammation or individuals with a more pronounced systemic immune response [34,35]. A subsequent evaluation of the same cohort has explored the performance of a panel of 1129 proteins, identifying 51 proteins that could predict CD with a 76% accuracy up to 5 years before diagnosis [39].

Similar findings have been found in the Nurses’ Health Study, where C-Reactive protein and IL-6 levels have been found to be increased in pre-diagnostic samples of IBD, although with a lower yield in those with UC [77]. This finding is consistent across different cohorts where the detection of subclinical inflammation in patients with UC has usually been underrepresented [78]. However, another international cohort—IBD Character Consortium—was able to identify MMP10, CXCL9, CCL11, SLAMF1, CXCL11, and MCP-1 as potential markers associated to preclinical UC using a 92-protein kit (Olink Bioscience, Uppsala, Sweden) [41]. Importantly, all these proteins, except MCP-1, were differentially regulated in an inception cohort compared with healthy controls. This is a rapidly evolving field where ongoing cohorts and biobank-based studies will be exploring potential biomarkers in both disease subtypes. Future investigations will also determine whether the differences observed between CD and UC are due to their respective pathogenic pathways or secondary to study methodology associated with the type of analysis performed and their timing. Nevertheless, international cohorts have clearly confirmed the presence of circulating biomarkers during the preclinical period, hence opening this field to new omics techniques that will allow better characterisation and integrative perspective of the initial processes preceding the onset of the disease.

### 3.2. Disease Prediction

Attention has turned to the use of microbial immune profiles, genomics, proteomics, and transcriptomics to develop prognostic tools. Profiles of antibodies to specific microbial determinants originally developed for diagnosis are also associated with worse CD disease outcomes [72]. However, this association may be confounded since these antibody responses increase with the length of disease duration [79]. New antibodies not associated with antimicrobial immune responses, including anti-integrin αvβ6 autoantibodies, act as both a pre-diagnostic and prognostic marker in UC, based on two independent incident cohorts that validated the findings [38]. Genome-wide association studies have identified prognostic gene loci, distinct from those associated with disease susceptibility, for both CD and UC; however, their relatively weak association makes them unsuitable for use in the clinical setting [80,81]. Efforts are now focused on polygenic risk scores as a prognostic tool rather than focusing on the diagnostic process [82].

Elevation of the inflammatory markers C-reactive protein and faecal calprotectin are indicative of ongoing inflammation and are associated with worse clinical outcomes, although arguably they are not true prognostic markers [83]. Advanced proteomic approaches are currently being applied in predictive models by several consortia and are discussed elsewhere in this review. Likewise, lipidomic profiles have potential roles in diagnosis and prognosis [84]. All these findings highlight how biomarkers are an area of extensive research with significant new advances. The RISK study [Risk Stratification and identification of Immunogenetic and microbial MarKers of rapid disease progression in children with Crohn’s disease] found risk prediction models based on transcriptomic signatures from intestinal tissue combined with clinical data predict stricturing disease; however, these findings require independent validation [85]. Transcriptome signatures in CD8+ T-cells in treatment-naive patients predict aggressive disease behaviour in IBD [73]. A total of 17 of the genes identified are included in a whole blood gene array [86] that is currently being evaluated in the predicting outcomes in CD using a molecular biomarker (PROFILE) randomised controlled trial [87]. If validated, this prognostic marker will represent the first commercially available tool with the potential to transform therapeutic approaches in newly diagnosed CD.

Early detection remains a challenge due to the absence of clinical manifestations. However, recent studies have uncovered molecular changes associated with the transition from a healthy-like to IBD-like physiology, starting at least 5 years before symptom onset [36,39,44]. Several initiatives are starting to chart the molecular changes that occur in the preclinical stage, which can potentially serve biomarker purposes. In this section, we provide an illustrative overview of three of these efforts and discuss relevant limitations in the area.

Since 2008, The Genetics, Environmental, Microbial (GEM) project has prospectively recruited healthy first-degree relatives (FDRs) of individuals with CD [88]. Molecular profiling of the FDRs that eventually developed IBD has provided important insights into the transition to disease. This includes a serum proteomic signature associated with future CD development [40] and increased intestinal permeability and serum levels of microbial antibodies 5 and 3 years before CD onset, respectively [36,40,44]. Regarding UC, proteolytic and elastase activity in pre-UC microbiota presents alterations similar to the ones found in UC-onset samples [46]. The Proteomic Evaluation and Discovery in an IBD Cohort of Tri-service Subjects (PREDICTS) has also provided new insights into the early immune alterations observed in the pathogenesis of IBD, from different omics analyses, including proteomics [39], serology [35,37,38], and metabolomic [42] signatures. Finally, IBD-Character is the third initiative that has provided relevant insights into IBD development. Through omics profiling of peripheral blood from patients at diagnosis, this collaboration has described transcriptional and epigenetic alterations with biomarker translational potential [41,89,90].

Although there are reasons for excitement, three important limitations hinder our progress toward identifying biomarkers for the preclinical stage. First, the role of the environment is rarely incorporated in preclinical studies. The evidence on early-life exposures [91] and smoking suggests that environmental factors are necessary to trigger the mechanisms that lead to disease [92,93]. Second, achieving proper sample sizes remains an issue. IBD studies are often limited by the number of recruited patients, and this is particularly relevant in preclinical studies. Some of the above-mentioned projects are showing promising results after 20 years of effort; however, large prospective cohorts from a multi-omics perspective are still needed in order to answer some of the key questions on the pathogenesis of the disease. Finally, there is a lack of validation of the most recent findings in this field, and they would need to undergo external replication in validated independent cohorts. Working towards ensuring the replicability of findings needs to be a top priority in the field.

## 4. Integration of Multiple Omics

Omics technologies have revolutionized our understanding of biological systems by enabling the large-scale study of pathogenic molecular components. Integrative multiple omics datasets are becoming increasingly important for gaining a comprehensive view of the biological processes involved in disease and can lead to the discovery of novel biomarkers and therapeutic targets. In this review, we have thoroughly addressed the current state of all the omics studies on preclinical IBD, highlighting the need for further research in this area. Despite the numerous advances made in recent years, there is still a long way to go before we fully understand the complex interaction between the different omics layers. Studies focused on multi-omics analyses such as the upcoming EARLY cohort (ClinicalTrials.gov: NCT05698745) will identify early biomarkers in preclinical IBD. To fully realize the potential of multi-omics for preclinical IBD, future studies must incorporate the wealth of emerging areas and techniques such as patient-derived organoids, comprehensive regulatory RNA profiling, and new artificial intelligence-based approaches.

The emerging focus on multi-omics profiling is a clear bright spot of the initiatives targeting the preclinical stage. The advances in high-throughput capabilities in genomics, transcriptomics, metabolomics, and the like allow for hypothesis-free inspection of a wide array of molecular layers in each individual [32,94]. By scanning several layers simultaneously, multi-omics approaches allow scaling-up and interrogation of molecular processes from several points of view and buttress the mechanistic inferences gained from single omics comparisons.

The aforementioned RISK study for paediatric CD exemplifies this point well. A multi-omics perspective uncovered a subpopulation of patients with upregulated expression of genes associated with the remodelling of the extracellular matrix, despite known serological and microbial markers not being greatly altered [85]. Although the landscape for preclinical IBD is still patchy, factors such as altered permeability, mucosal damage, and increased inflammation are emerging as prodromal components of the early stages of the disease [5,39]. Multi-omics studies will be needed to put these pieces together, clarify the timing of action, and, eventually, devise ways of inspecting their specific relevance in each patient.

An often-ignored aspect of multi-omics approximations is particularly relevant for preclinical IBD. Multi-omics studies are the way to go beyond basic identification of culprits, and to propose mechanisms that lead to disease. However, inferring causality purely from multi-omics data may not be possible. The transition to disease has a large impact on body physiology, and hence the development of disease induces many alterations in cellular processes that are picked up in the omics profiles of patients [95,96]. This leads to a problem that resembles the need to distinguish between “drivers” versus “passengers” in cancer studies. Indeed, due to the progressive build-up of physiological alterations suffered by patients, the differences between “cause” and “consequence” are less clear as we incorporate patients who are at more advanced stages of the natural cause of the disease. The focus on characterising preclinical patients who are identified in the early stages of the disease is, therefore, a good tactic for maximizing the ability to identify genuine trigger mechanisms that are useful for biomarker and prediction purposes.

In multi-omics efforts, a diverse range of experts, including statisticians and bioinformaticians, are needed to ‘squeeze’ the data, collaborations are key to replicate findings across cohorts and provide room for meta-analyses, and the clinician perspective would be needed to integrate the messages and maximize the chances of translational impact. Rather than simplistic ambitions for new “signatures with biomarker potential”, the goal of emerging integrative multi-omics initiatives should be to carefully clarify the causal regulatory cascade that finally leads to IBD. Ongoing cohorts have provided many new details about the early stages of IBD, but future prospective studies with a multidisciplinary and integrative perspective will help in revealing new insights that will lead to a better understanding of IBD.

## Figures and Tables

**Table 1:** Summary of the main multi-omics findings in preclinical inflammatory bowel disease.

Study/Cohort	Type and Number of Individuals	Type of IBD	Type of Samples	Omics	Main Findings
Malmö Diet and Cancer cohort	72 UC 140 healthy controls 37 healthy twins	UC	Blood, dried blood spots	Proteomics	Proteomic profile found to be upregulated years before UC diagnosis [41]
Swedish Newborn Dry Blood Spots cohort					
Swedish IBD Twin cohort					
Northern Sweden Health and Disease Study					
The Crohn’s and Colitis Canada Genetics, Environmental, Microbial (GEM) Project	5122 first-degree relatives	UC CD	Blood, stool, urine	Multiomics	Genetic signature of FDR [47] Proteomics signature [40] Microbiome composition and gut barrier dysfunction in FDR [45] Faecal biomarkers [46] Serological findings with antimicrobial antibodies [36] Altered intestinal permeability [44] Anti-integrin antibodies [38]
Multiplex families, Road to Prevention cohort	38 multiplex families	UC CD	Blood, stool, teeth, hair, saliva	Multiomics	Affected siblings are likely consecutively distributed [48]
TWIN cohort	124	UC CD	Blood, urine, stool, oral, rectal biopsies	Multiomics	Microbiome signatures [43]
PREDICTS cohort	1000 UC 1000 CD 500 Healthy controls	UC CD	Blood	Proteomics Serology	Protein signatures [39] Antimicrobial antibodies [34,35] Anti-GM-CSF antibodies [37] Anti-integrin antibodies [38]
Nurses’ Health Study	104 IBD	UC CD	Urine	Metabolome	Altered prediagnostic metabolites and metabolic pathways [42]

CD: Crohn’s disease; IBD: inflammatory bowel disease; FDR: first-degree relatives; GM-CSF: Granulocyte-Macrophage Colony-Stimulating Factor; UC: ulcerative colitis.

## Abbreviations

IBD	inflammatory bowel disease
CD	Crohn’s disease
UC	ulcerative colitis
GM-CSF	Granulocyte-Macrophage Colony-Stimulating Factor
ASCA	Anti-Saccharomyces cerevisiae antibodies
pANCA	perinuclear anti-neutrophil cytoplasmic antibodies
ACCA	Antichitobioside antibodies
OmpC	Outer membrane proteins
